# Cannabidiol and Cannabidiol Metabolites: Pharmacokinetics, Interaction with Food, and Influence on Liver Function

**DOI:** 10.3390/nu14102152

**Published:** 2022-05-21

**Authors:** Kieran Shay Struebin Abbotts, Taylor Russell Ewell, Hannah Michelle Butterklee, Matthew Charles Bomar, Natalie Akagi, Gregory P. Dooley, Christopher Bell

**Affiliations:** 1 Department of Health and Exercise Science, Colorado State University, Fort Collins, CO 80523-1582, USA; kieran.abbotts@colostate.edu (K.S.S.A.); taylor.ewell@colostate.edu (T.R.E.); hmbutter@rams.colostate.edu (H.M.B.); matt.bomar@rams.colostate.edu (M.C.B.); 2 Department of Environmental and Radiological Health Sciences, Colorado State University, Fort Collins, CO 80523-1680, USA; natalie.akagi@gmail.com (N.A.); gregory.dooley@colostate.edu (G.P.D.)

**Keywords:** cannabis, cannabinoid, metabolism, thermogenesis, energy expenditure, pharmacodynamics, insulin, triglyceride

## Abstract

Cannabidiol (CBD) is widely available and marketed as having therapeutic properties. Over-the-counter CBD is unregulated, many of the therapeutic claims lack scientific support, and controversy exists as to the safety of CBD-liver interaction. The study aims were to compare the pharmacokinetics of commercial CBD and CBD metabolites following the ingestion of five different CBD formulations, determine the influence of CBD on food induced thermogenesis, determine the influence of food on CBD pharmacokinetics, and determine the influence of CBD on markers of liver function. Fourteen males (body mass index ≥ 25 kg/m^2^) were studied in a placebo-controlled, randomized, crossover design. On five occasions, different CBD formulations were ingested (one per visit). On two additional occasions, CBD or placebo was ingested following a meal. CBD servings were standardized to 30 mg. Considerable pharmacokinetic variability existed between formulations; this pharmacokinetic variability transferred to several of the metabolites. CBD did not influence food induced thermogenesis but did favorably modify early insulin and triglyceride responses. Food appreciably altered the pharmacokinetics of CBD. Finally, CBD did not evoke physiologically relevant changes in markers of liver function. Collectively, these data suggest that consumers should be aware of the appreciable pharmacokinetic differences between commercial CBD formulations, CBD is unlikely to influence the caloric cost of eating but may prove to be of some benefit to initial metabolic responses, consuming CBD with food alters the dynamics of CBD metabolism and increases systemic availability, and low-dose CBD probably does not represent a risk to normal liver function.

## 1. Introduction

Cannabidiol (CBD), a non-psychotropic component of *Cannabis sativa* L., is regularly consumed by millions of people, in part because of the belief that it bestows a variety of health benefits [1]. Based on recent reviews, it appears that CBD may indeed bestow some health benefits, but in general the claims likely outweigh the supporting evidence [2,3]. In this regard, empirical research may have unintentionally contributed to the confusion associated with the purported benefits of CBD on account of widely discrepant findings. There are many potential explanations for these discrepancies, not the least of which being the number of possible ways by which to consume CBD (such as ingestion, inhalation, transdermal, sublingual, and colonic) and the resultant variability in systemic CBD availability [4]. Even when the route of administration is standardized, systemic CBD availability varies greatly. Previously, we [5] and others [6,7] have described considerable differences in the pharmacokinetics of ingestible CBD. These differences can be attributed to the CBD dose [8], whether the dose is co-administered with or without food [8,9,10], and/or the composition of the CBD formulation [7,11,12]. With respect to the latter, advances in the engineering of dietary supplements have led to the development of strategies for accelerating the transport of ingested CBD from the gut to the circulation, avoiding first-pass (hepatic) metabolism, and/or limiting CBD breakdown during first pass metabolism [7,11,12]. In the current study, we have extended our previous work to compare different strategies for promoting CBD availability and improving CBD pharmacokinetics. These strategies include the presence/absence of different excipients (including sorbitol and maltodextrin), the comparison of water- and lipid-soluble preparations, and the comparisons of gum arabic and medium chain triglyceride (MCT) coconut oil. The first aim of the current study was to compare the pharmacokinetics of cannabidiol (CBD) and CBD metabolites (6-OH-CBD, 7-OH-CBD, and CBD-COOH) following ingestion of five different CBD formulations. We hypothesized that, when delivered in an aqueous beverage, water-soluble emulsions would prove superior to lipid-soluble preparations with respect to maximal circulating CBD concentration (C_max_) and time to maximal circulating CBD concentration (t_max_).

Obesity and the overweight state have become a worldwide problem [13]. Excessive adiposity is associated with an increased risk of developing chronic cardio-metabolic diseases and, of recent importance, an increased likelihood of adverse complications stemming from coronavirus disease 2019 (COVID-19) [14,15]. Long term positive energy balance (where energy expenditure is less than energy intake) is thought to contribute to excess adiposity. The influence of CBD on the regulation of energy expenditure as it relates to energy balance and weight gain has received relatively little attention. In animal models of obesity, and in selected studies of humans with excess adiposity, the endocannabinoid system is upregulated and is thought to contribute to an unfavorable metabolic phenotype [16,17,18,19]. CBD inhibits the actions of cannabinoid receptors (CB1 and CB2) in a manner characteristic of an inverse agonist/negative allosteric modulator [20,21]. Accordingly, it is plausible, from a metabolic perspective, that CBD may also favorably modify the endocannabinoid system in humans [16]. At present, the influence of CBD on diet-induced thermogenesis in adult humans with overweight and obesity is unclear. The second aim of the current study was to explore the novel hypothesis that acute administration of CBD would increase the thermic effect of food, an important contributor to total daily energy expenditure [22].

The experimental approach for our second aim pertaining to the thermic effect of food provided us with a unique opportunity to simultaneously determine the influence of a meal on CBD pharmacokinetics. Ingestion of a mixed macronutrient meal prior to CBD ingestion will promote the release of various digestive chemicals and liquids, including bile, that may influence the transport of CBD from the gut and to the circulation [8,9]. The third aim of the current study was to compare CBD pharmacokinetics when the CBD was ingested in the fasted and fed state. We hypothesized that prior feeding would slow t_max_ but increase C_max_ and systemic CBD availability.

Finally, one area of significant controversy pertaining to the potential health benefits of CBD relates to the liver [23]. While some studies have suggested that CBD may promote favorable liver function, and may even be included in comprehensive treatments for liver diseases [24], others have suggested that CBD may harm the liver [25]. An additional goal of the current study was to determine the acute influence of a variety of different CBD formulations on circulating markers of liver function, and to determine if CBD availability (i.e., circulating concentration) predicts the magnitude and change of these circulating markers.

## 2. Materials and Methods

The study was registered as a clinical trial (ClinicalTrials.gov Identifier: NCT04971837, accessed on 22 July 2021). The protocol was conducted according to the guidelines of the Declaration of Helsinki and was approved by the Institutional Review Board of Colorado State University (Protocol #21-10634H, 24 March 2021). All participants provided written informed consent prior to beginning the study.

### 2.1. Participants

Adult males and females were invited to participate. Inclusion criteria consisted of age 18 years or older, body mass greater than 50 kg, body mass index greater than or equal to 25 kg/m^2^ (thereby satisfying the criteria for overweight or obesity), absence of any known metabolic or gastrointestinal disease, and willingness to refrain from all products derived from *Cannabis sativa* L., including CBD, 72 h prior to each study visit. Exclusion criteria included pregnancy, breast-feeding, previously identified food allergies, disorders of the autoimmune system, celiac disease, inflammatory bowel disease, or gastrointestinal cancers. In addition, to avoid potential confounders and/or unfavorable interactions with CBD, adults who reported regularly or recently using any of the following medications were not permitted to enroll: steroids, 3-hydroxy-3-methylglutaryl coenzyme A (HMG-CoA) reductase inhibitors, calcium channel blockers, antihistamines, human immunodeficiency virus (HIV) antivirals, immune modulators, benzodiazepines, antiarrhythmics, antibiotics, anesthetics, antipsychotics, antidepressants, anti-epileptics, beta adrenergic receptor blockers, proton pump inhibitors, non-steroidal anti-inflammatory drugs (NSAIDs), angiotensin receptor II blockers, oral hypoglycemic agents, and sulfonylureas. Finally, anyone who reported experiencing a prior adverse reaction to ingesting products derived from *Cannabis sativa* L. were excluded from participation.

### 2.2. Protocol Overview

Participants were required to visit the laboratory on eight separate days. The first visit entailed a review of medical history and assessment of body composition. The remaining seven visits incorporated a randomized, placebo-controlled, repeated measures crossover design. Prior to arrival at the laboratory, participants abstained from all food and beverages (except water) for 12-h and avoided vigorous physical activities for 24-h. As per previous studies of CBD pharmacokinetics [5,26], to facilitate negligible baseline circulating CBD concentrations, every visit was preceded by a minimum 72-h abstention from all products derived from *Cannabis sativa* L., including CBD. Accordingly, each study visit was separated by a minimum of 72-h. The time of arrival at the laboratory was kept constant for each participant (±1 h).

Two of the laboratory visits began with a measurement of the resting metabolic rate. Participants then consumed a mixed macronutrient meal followed immediately by ingestion of either CBD or placebo. Over the next 4-h, energy expenditure was measured intermittently, and venous blood was sampled. Five of the laboratory visits comprised ingestion of different CBD formulations (one per visit). Each serving was standardized to 30 mg of CBD. Prior to and following CBD ingestion, venous blood was sampled over 4 h.

### 2.3. Procedures

The initial screening visit began with written informed consent, and an interview and questionnaire to obtain medical history. This was followed by measurement of height and body mass for the calculation of body mass index, and assessment of body composition via dual energy X-ray absorptiometry (Hologic, Discovery W, QDR Series, Bedford, MA, USA) as reported in previous studies [27,28].

The two visits involving ingestion of a meal were identical in all aspects except for the administration of either CBD or placebo. Resting metabolic rate and the thermic effect of food were determined using a modification of previously described procedures [29,30]. Study participants were prepared for the measurement of heart rate and blood pressure (automated device; IntelliVue MP5 Patient Monitor, Philips Healthcare, Andover, MA, USA) and a venous catheter was placed in an antecubital vein. Following instrumentation, resting metabolic rate was measured over 45 min; the first 15 min were considered a habituation period and were excluded from analysis. Oxygen uptake (VO_2_) and carbon dioxide production (VCO_2_) were averaged each minute, over 30 min, using a ventilated hood indirect calorimetry system (Parvo Medics TrueOne 2400, Salt Lake City, UT, USA). Respiratory exchange ratio, a crude indicator of substrate utilization, was calculated as VCO_2_/VO_2_. The metabolic rate was calculated using the Weir equation [31]. Following the measurement of the resting metabolic rate, participants imbibed a commercially available liquid mixed meal (Boost Balanced Nutrition Drink; Nestle Health and Science, Fremont, MI, USA; 22% fat, 62% carbohydrate, 16% protein). To standardize the stimulus, the administered caloric load was equivalent to 40% of the resting metabolic rate. Based on previous studies [29,30], this is thought to represent approximately 30% of total daily caloric requirements and is reflective of a normal meal. Participants consumed the liquid meal within 10 min. Immediately following meal ingestion, participants were administered either 30 mg of CBD (formulation 725; see below for details) or placebo in 227 mL (8 oz) of water. Energy expenditure was measured over 25 min of the next eight consecutive 30-min periods (i.e., 4-h), allowing participants brief relief (5 min of each half hour) from the ventilated hood. During these measurements, participants were required to rest, but not sleep, in a semi-recumbent position. The use of electronic devices such as telephones and laptops was not permitted. The room in which data collection occurred was quiet, dimly lit and temperature controlled (~22 °C).

Venous blood (~9 mL/sample) was collected for subsequent analysis of circulating concentrations of CBD and CBD metabolites prior to (i.e., at baseline during the measurement of resting metabolic rate), and 10, 20, 30, 45, 60, 120, 180, and 240 min after CBD ingestion. Venous blood (~3 mL/sample) was also sampled for subsequent analysis of circulating concentrations of markers of liver function prior to, and 60, and 240 min after CBD ingestion. Finally, venous blood (~3 mL/sample) was also sampled for the measurement of circulating glucose, insulin, and triglyceride concentrations prior to, and 10, 20, 30, 45, 60, 90, 120, 150, 180, 210 and 240 min after food and CBD/placebo ingestion.

The five visits not involving administration of a meal were identical in all aspects except for the specific CBD preparation that was to be ingested. On arrival, a venous catheter was introduced to an antecubital vein. Venous blood (~9 mL) was collected for subsequent analysis of circulating concentrations of CBD and CBD metabolites prior to, and 10, 20, 30, 45, 60, 120, 180, and 240 min after CBD ingestion. Venous blood (~3 mL) was also sampled for analysis of circulating concentrations of markers of liver function prior to, and 60 and 240 min after CBD ingestion.

### 2.4. CBD Preparations

The key features of each of the CBD formulations are presented in Table 1. All CBD was provided by Caliper Foods (Commerce City, CO, USA). Five CBD formulations were compared. Each serving contained 30 mg of CBD. Thirty mg is a common dose for commercially available, over-the-counter CBD products, and is the serving size recommended by the manufacturer of the products studied in the current investigation. Formulation 725 was water soluble and contained sorbitol, a low-calorie sugar alcohol commonly used as an excipient in the preparations of many pharmaceuticals [32]. Formulation 088 was an MCT coconut oil-based tincture; MCTs are considered an industry standard for dietary supplements, including CBD [33] and curcumin [34]. Formulation 126 was water soluble and comprised a 10% CBD gum arabic mixture with a tapioca maltodextrin base. Maltodextrin is thought to help favorably modify osmotic pressure and promote transport from the intestine [35]. Formulation 213 was similar to formulation 126 in that it was water soluble and comprised a 10% gum arabic mixture, but maltodextrin was replaced with sorbitol. Finally, formulation 625 was a CBD isolate, not water soluble (i.e., hydrophobic), and was comprised of a pure form of CBD as a crystalline powder with >99% purity. This formulation did not contain an emulsifier or excipient. All formulations were administered in 227 mL (eight oz) of water and were consumed within less than 30 s of administration.

### 2.5. Blood Handling and Analysis

Fresh blood for CBD, CBD metabolite, and insulin analysis was transferred to chilled tubes containing ethylenediaminetetraacetic acid (EDTA). Blood to be analyzed for markers of liver function, and for triglycerides, was immediately transferred to chilled tubes containing lithium heparin. Plasma aliquots (1 mL) were separated from each of the samples and stored at −70 °C for later analysis. Finally, whole blood to be used for the determination of glucose concentration was analyzed immediately using an automated device (YSI 2900 STAT Glucose Lactate Analyzer, YSI Inc., Yellow Springs, OH, USA).

Plasma insulin concentration was determined in triplicate via enzyme-linked immunosorbent assay ((ELISA) Crystal Chem, Inc., Elk Grove Village, IL, USA). An automated device (Piccolo, Abaxis, Inc., Union City, CA, USA) was used to analyze plasma for markers of liver function, and for triglyceride concentration. Markers of liver function are often coupled with markers of kidney function. In this regard, our automated analyzer also combined liver with kidney markers. Collectively, the markers were calcium, blood urea nitrogen, creatinine, alkaline phosphatase, alanine, aminotransferase, aspartate aminotransferase, total bilirubin, albumin, and total protein.

### 2.6. CBD Reagents and Supplies

CBD, 7-hydroxy-CBD, 7-carboxy-CBD, 7-hydroxy-CBD-D3, and 7-carboxy-CBD-D3 were obtained from Cerilliant (Round Rock, TX, USA). CBD, 7-hydroxy-CBD, 6-hydroxy-CBD was also obtained from Cayman Chemical (Ann Arbor, MI, USA). Water, methanol, and acetonitrile (LC–MS grade) were procured from Millipore (Burlington, MA, USA). Dansyl chloride, sodium bicarbonate, sodium carbonate, acetic acid, and formic acid (LC-MS-grade) were bought from Sigma-Aldrich (St. Louis, MO, USA). Captiva EMR-Lipid columns (1 mL, 40 mg) were acquired from Agilent Technologies (Santa Clara, CA, USA). Chromatography was performed with a Restek raptor biphenyl column (2.1 × 100 mm, 5 μm) purchased from Restek Inc. (Bellefonte, PA, USA).

### 2.7. Calibrators, Quality Controls, and Internal Standard Preparation

Volumes of methanolic stock standard mixtures were added to 200 μL of cannabinoid free plasma to create matrix matched calibrators and controls. Working standard mixes containing 0.01, 0.1, or 1.0 μg/mL of CBD, 6-hydroxy-CBD, 7-hydroxy-CBD and 7-carboxy-CBD were created using stock standards procured from Cayman Chemical or Cerilliant. These mixes were then used to create calibrators for CBD, 7-hydroxy-CBD, and 6-hydroxy-CBD at 0.1, 0.25, 0.5, 1, 5, 10, and 50 ng/mL. Calibrators for 7-carboxy-CBD were created at 0.25, 0.5, 1, 5, 10, 50, and 100 ng/mL. Quality control samples were created at 1, 10, and 25 ng/mL for each analyte. These control standards were created from stock standard obtained from different vendors, if available, to confirm the calibrator stock standards. After every 20 research participant samples, quantity control samples were ran with an expected accuracy of +/− 20%. The internal standard mix solution contained 300 ng/mL CBD-D3, 300 ng/mL 7-hydroxy-CBD-D3, and 800 ng/mL 7-carboxy-CBD-D3 in methanol.

### 2.8. CBD Analysis by Liquid Chromatography Tandem Mass Spectrometry (LC-MS/MS)

Protein precipitation, lipid removal, and derivatization with dansyl chloride (Supplementary Table S1) were used to prepare plasma samples and matrix matched standards and quality controls for LC-MS/MS analysis. 10 μL of internal standard solution was added to 200 μL of plasma and the sample was mixed in a microcentrifuge tube. 1 mL of acetonitrile was added and vortexed for 30 s to precipitate proteins. Samples were centrifuged and supernatants transferred to Captiva EMR-Lipid columns for lipid removal. Using a positive pressure manifold, 3 psi of pressure was applied to the samples to elute through columns. Eluents were collected into a clean glass test tube and dried to approximately 100 μL under nitrogen at a temperature of 40 °C prior to derivatization. Eluents were then mixed with 50 μL of 5 mg/mL dansyl chloride in acetonitrile, 100 μL of a 0.1 M sodium carbonate-bicarbonate buffer (pH 10) and relocated to autosampler vials fitted with 400 μL glass inserts. Samples were incubated at 55 °C for 10 min to derivatize the analytes, cooled to room temperature, and neutralized with 10 μL of acetic acid prior to LC-MS/MS analysis. Samples were analyzed with an Agilent 1290 UHPLC that was coupled to an Agilent 6460 triple quadruple mass spectrometer that in turn was equipped with an Agilent Jet Stream electrospray ionization source (Agilent, Santa Clara, CA, USA). Cannabinoids were first chromatographically separated on a Restek raptor biphenyl column (2.1 × 100 mm, 5 μm) held at 40 °C. A sample volume of 10 μL was injected and a mixture of water with 0.1% formic acid (A) and methanol with 0.1% formic acid (B) at a flow rate of 0.4 mL/min. The gradient elution started at 60% B, increasing to 80% B at 1.5 min, finishing to 100% B at 4.5 min. The ionization source conditions used were as follows: positive polarity, nebulizer 35 psi; gas flow of 12 L/min at 300 °C; sheath gas flow of 12 L/min at 385 °C; capillary voltage of 3500 V; nozzle voltage of 500 V. The ion transitions monitored are displayed in Table S1. Analytes were confirmed by retention time and the product ion ratio correlation between the sample peaks and corresponding standards (±20%). The data collection and processing were performed by using Agilent MassHunter Quantitative software (v.B.08.01). Quantitation was performed with linear regression using 7-point calibration curves from 0.1 ng/mL to 50 ng/mL for CBD, 7-hydroxy-CBD, and 6-hydroxy-CBD. A 7-point calibration curves from 0.25 ng/mL to 100 ng/mL was used for 7-carboxy-CBD.

### 2.9. Pharmacokinetic Analysis

Dedicated software (Phoenix WinNonlin v8.3, Certara, NJ, USA) was used to perform pharmacokinetic analysis of the circulating concentrations of CBD and the CBD metabolites for each of the preparations. Values below the limit of quantitation were classified as “missing” for non-compartmental analysis. The trapezoidal method was used to calculate areas under the CBD concentration curves. Derived pharmacokinetic parameters included t_max_, C_max_, the area under the curve representing total exposure between 0 and 4 h (AUC_0–4_), an estimate of the total exposure over time (AUC_0–inf_), the amount of time taken to decrease the circulating concentration to half of the maximal value (t_½_), the rate of removal from the body (k_e_), and the volume of distribution, an estimate of the degree of distribution in the body tissue vs. the plasma (V_d_).

### 2.10. Statistical Analysis

All data, unless otherwise stated, are presented as mean and standard deviation. Statistical analyses were performed using dedicated, commercially available software (SigmaStat for Windows 3.5, Systat Software, Inc., Chicago, IL, USA). Differences in pharmacokinetic parameters between CBD formulations for CBD, and the CBD metabolites were explored using one-way analysis of variance (ANOVA) with repeated measures. Tukey’s tests were used to further examine the identified main effects. The thermic effect of food was examined using two-way ANOVA with repeated measures comparing energy expenditure across time for the two conditions (i.e., CBD and placebo); respiratory exchange ratio and circulating concentrations of glucose, insulin, and triglycerides were compared in the same way. The thermic effect of food was also examined by comparing the area under the curve (trapezoidal method) as previously described [29,30,36]. Circulating markers of liver and kidney function were compared across time and between CBD formulations using two-way ANOVA with repeated measures and post-hoc Tukey's tests when appropriate. Relations between circulating CBD concentrations and markers of liver and kidney function were explored using Pearson correlations. The level of statistical significance was set at *p* < 0.05.

## 3. Results

### 3.1. Participants

**Figure 1:**
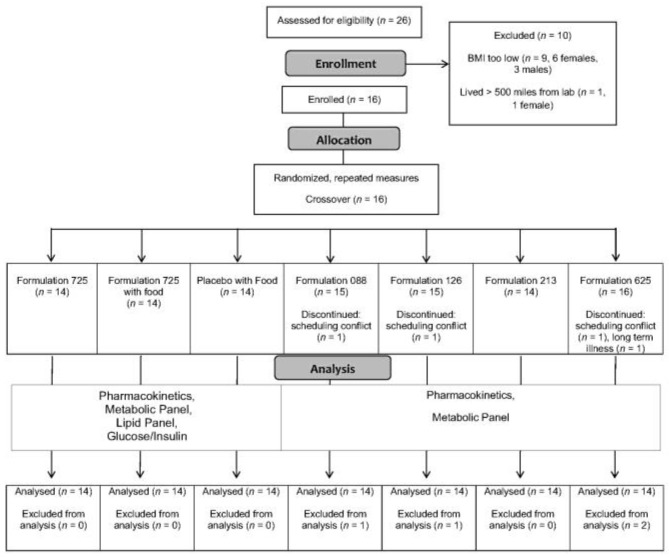
Consolidated Standards of Reporting Trials (CONSORT) flow diagram. Abbreviation: BMI body mass index.

The progress of the participants throughout the study (from screening and enrollment through to completion) is displayed in Figure 1. Twenty-six adults responded to study recruitment efforts, nine of whom failed to satisfy inclusion criteria (body mass index lower than threshold) and one who was excluded for logistical reasons (lived more than 500 miles from the laboratory). Sixteen adults were enrolled but two were unable to complete the entire protocol, one due to recurring long-term illness and the other due to repeated scheduling conflicts. Fourteen adults, all males, completed the study. Selected physiological characteristics are provided in Table 2. Consistent with the inclusion and exclusion criteria, the physiological characteristics were unremarkable, and were as expected for males with overweight or obesity who were otherwise healthy.

### 3.2. CBD, CBD Metabolites and Pharmacokinetics

**Figure 2:**
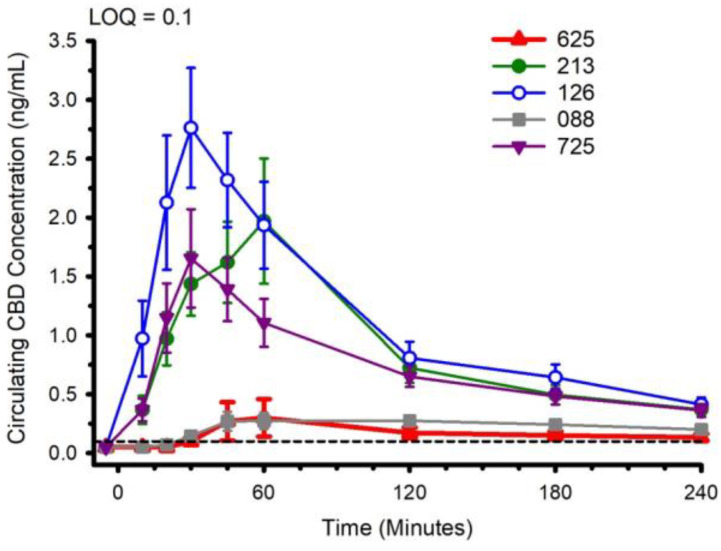
Circulating cannabidiol (CBD) concentration following ingestion of each of the CBD formulations. LOQ: Limit of Quantitation. Data: Mean and standard error.

**Figure 3:**
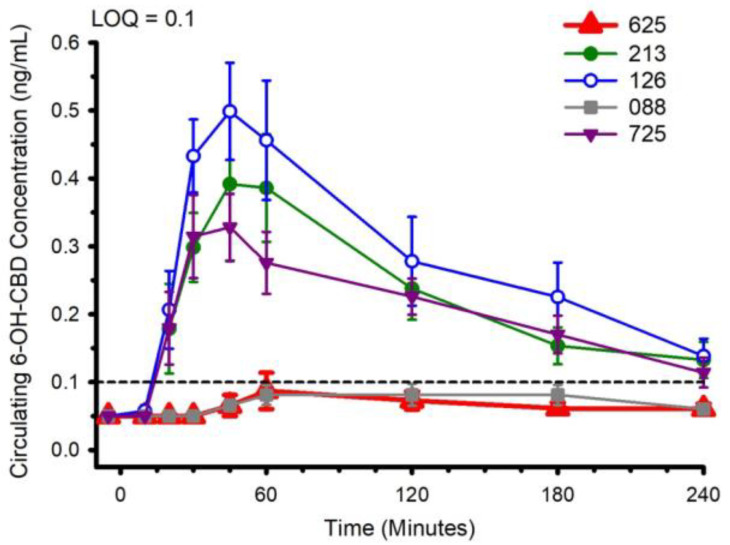
Circulating 6-OH-CBD concentration following ingestion of each of the cannabidiol formulations. Each CBD serving was standardized to 30 mg. LOQ: Limit of Quantitation. Data are mean and standard error.

**Figure 4:**
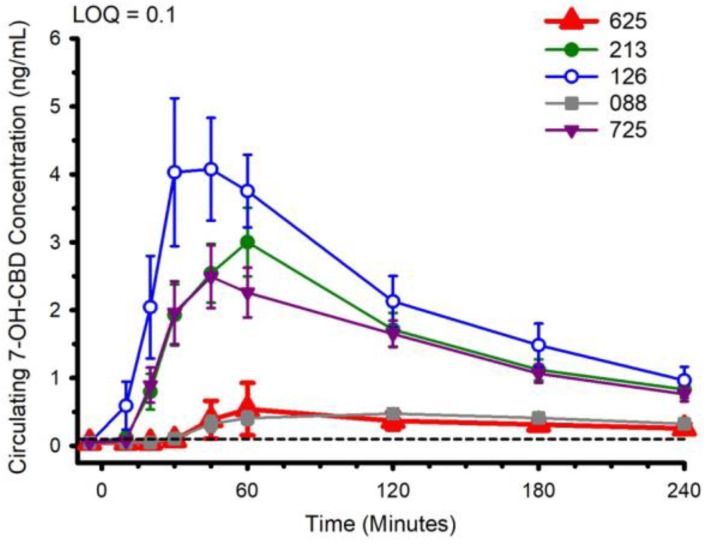
Circulating 7-OH-CBD concentration following ingestion of each of the cannabidiol formulations. Each CBD serving was standardized to 30 mg. LOQ: Limit of Quantitation. Data are mean and standard error.

**Figure 5:**
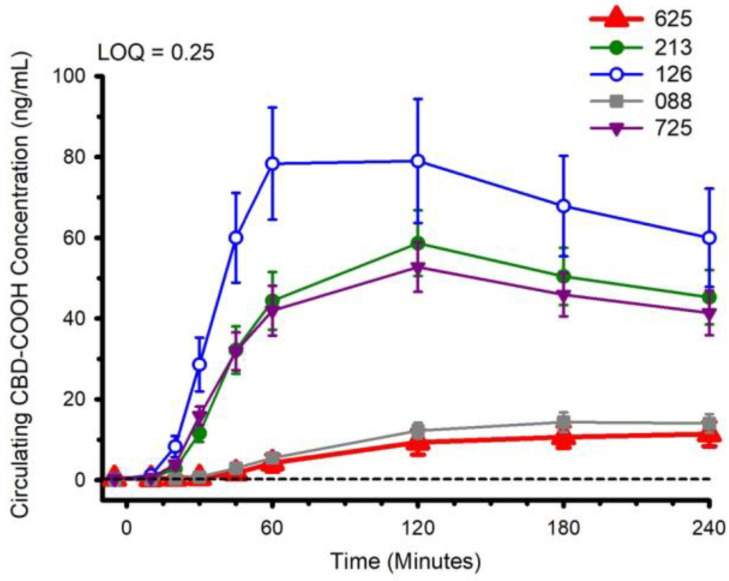
Circulating CBD-COOH concentration following ingestion of each of the cannabidiol formulations. Each CBD serving was standardized to 30 mg. LOQ: Limit of Quantitation. Data are mean and standard error.

Circulating concentrations of CBD, and CBD metabolites 6-OH-CBD, 7-OH-CBD, and CBD-COOH are presented in Figure 2, Figure 3, Figure 4 and Figure 5. The pharmacokinetic data for CBD, 6-OH-CBD, 7-OH-CBD, and CBD-COOH are presented in Table 3, Table 4, Table 5 and Table 6, respectively. It was not possible to calculate some pharmacokinetic parameters (i.e., AUC_inf_, t_½_, k_e_ and V_d_) for all of the CBD formulations on account of insufficient values above the limit of quantitation during the first hour of blood collection and/or the inability to capture the full elimination profile. Baseline (Time 0) concentrations for all CBD formulations, for all participants, were below the limit of quantitation (0.1 ng/mL), suggesting that sufficient washout between trials was provided.

The water-soluble preparations appeared to display superior CBD pharmacokinetics. The fastest values returned for t_max_ were from formulations 126 and 725, and the greatest C_max_ values were from formulations 126 and 213. With respect to the measured metabolites, in general, the same differences observed in circulating CBD pharmacokinetics carried over to the pharmacokinetics of the metabolites. The highest values for C_max_ and AUC_0–4_ and fastest values for t_max_ were typically observed in formulation 126; the lowest and slowest values were reported for the hydrophobic formulations, 088 and 625. Although not a stated aim of the current investigation, we were able to determine the relations between parameters of body composition and CBD pharmacokinetic parameters using Pearson correlations. Consistent with previous reports [5], none of the body composition parameters were consistently related to any of the CBD pharmacokinetic parameters across all formulations.

### 3.3. CBD and The Thermic Effect of Food

**Figure 6:**
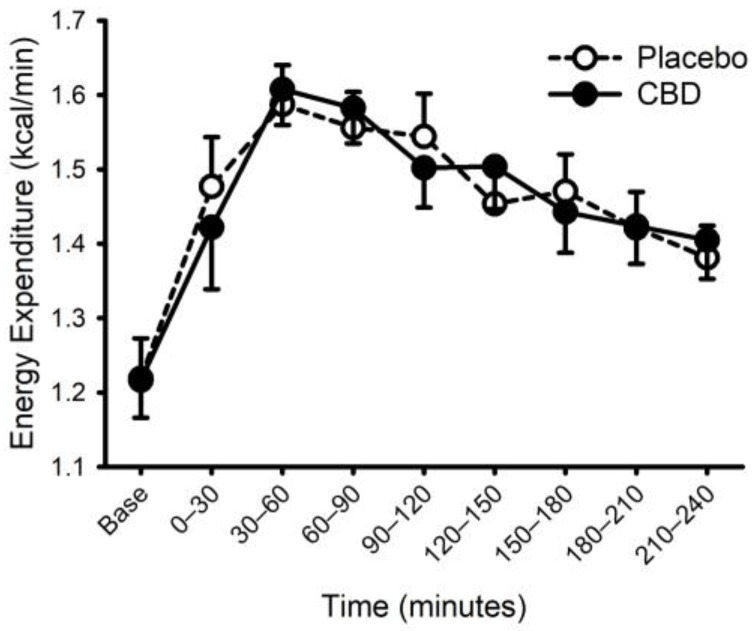
Energy expenditure prior to and following consumption of a mixed macronutrient meal and placebo or 30 mg of cannabidiol (CBD). Food ingestion increased energy expenditure (main effect of time; *p* < 0.001); CBD did not alter the response (placebo vs. CBD × time interaction; *p* = 0.32). Data are mean and standard error.

**Figure 7:**
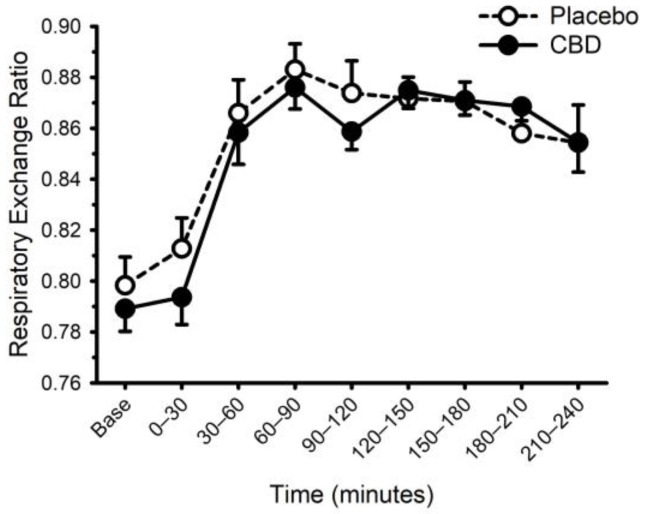
Respiratory exchange ratio prior to and following consumption of a mixed macronutrient meal and placebo or 30 mg of cannabidiol (CBD). Respiratory exchange ratio was increased after food (main effect of time: *p* < 0.001); CBD did not alter this response (placebo vs. CBD × time interaction; *p* = 0.13). Data are mean and standard error.

Energy expenditure and respiratory exchange ratio, prior to and following ingestion of food with or without CBD, are presented in Figure 6 and Figure 7. Prior to food plus placebo or CBD ingestion, the resting metabolic rate was similar (1755 ± 290 vs. 1752 ± 272 kcal/day; *p* = 0.93), as was the respiratory exchange ratio (0.80 ± 0.04 vs. 0.79 ± 0.03; *p* = 0.31). As expected, food ingestion increased energy expenditure (main effect of time; *p* < 0.001) but CBD did not alter this response (placebo vs. CBD × time interaction; *p* = 0.32). Consistent with these data, CBD did not influence the area under the response curve for energy expenditure (Placebo: 58 ± 21 vs. CBD: 59 ± 14 ((kcal/min) × min); *p* = 0.87). Similarly, while the respiratory exchange ratio was increased after food (main effect of time: *p* < 0.001), CBD did not alter this response (placebo vs. CBD × time interaction; *p* = 0.13).

Heart rate and systolic blood pressure were increased above resting values 30-min after food ingestion and remained greater throughout the 4-h of data collection (Supplementary Figures S1 and S2; main effect of time both *p* < 0.001). CBD did not influence the heart rate or systolic blood pressure response (placebo vs. CBD × time interaction; both *p* > 0.40). Diastolic pressure was greater at minute 240 compared with baseline (*p* = 0.003); CBD did not influence this response (placebo vs. CBD × time interaction *p* = 0.07).

**Figure 8:**
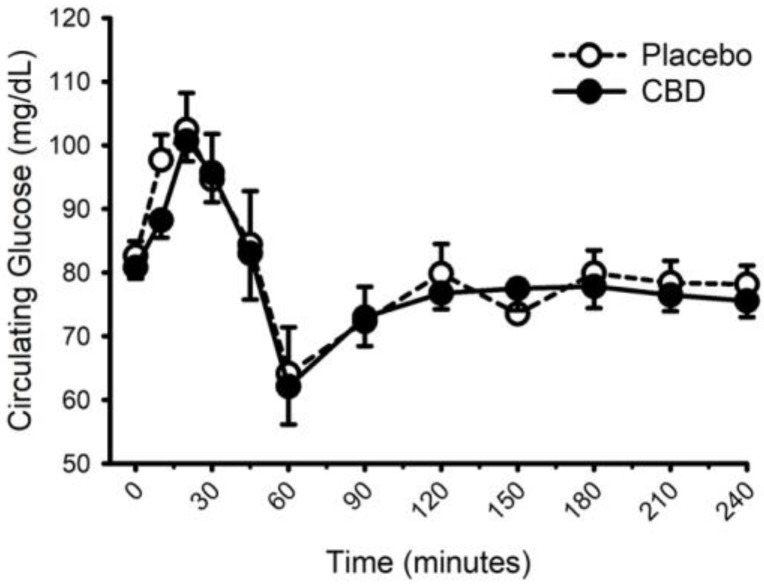
Circulating glucose concentration prior to and following consumption of a mixed macronutrient meal and placebo or 30 mg of cannabidiol (CBD). CBD did not influence the glucose response to food (placebo vs. CBD × time interaction; *p* = 0.31). Data are mean and standard error.

**Figure 9:**
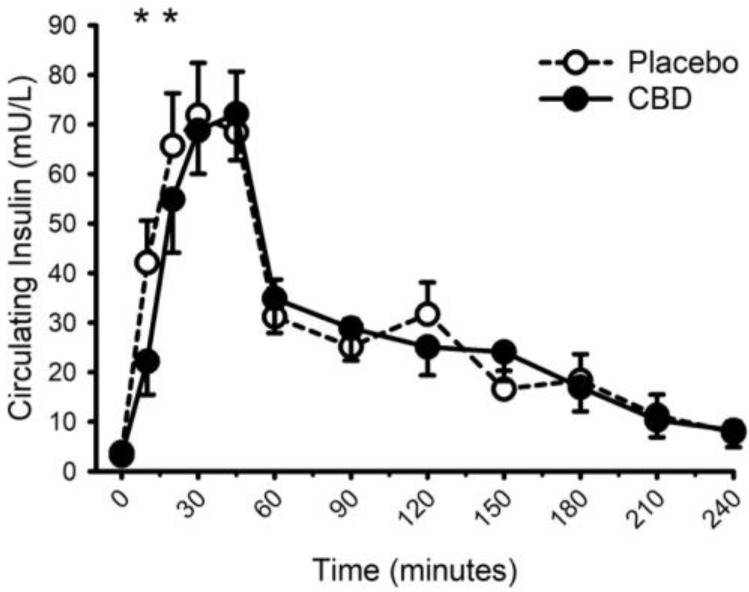
Circulating insulin concentration prior to and following consumption of a mixed macronutrient meal and placebo or 30 mg of cannabidiol (CBD). CBD evoked lower insulin concentrations at minutes 10 and 20 (placebo vs. CBD × time interaction; *p* = 0.013 indicated as *). Data are mean and standard error.

**Figure 10:**
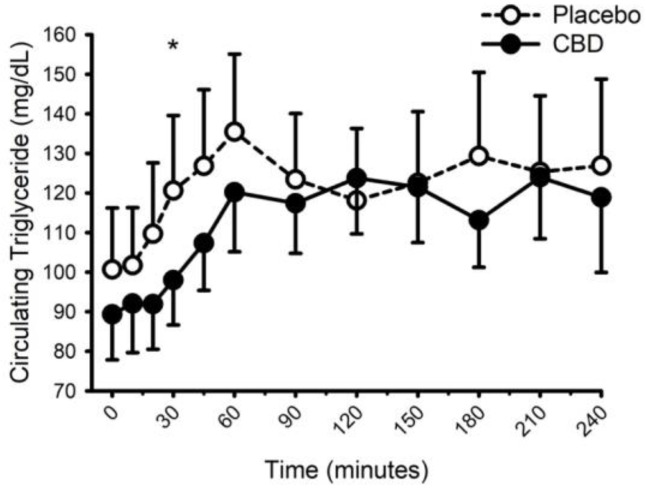
Circulating triglyceride concentration prior to and following consumption of a mixed macronutrient meal and placebo or 30 mg of cannabidiol (CBD). CBD evoked lower triglyceride concentrations at minute 30 (placebo vs. CBD × time interaction; *p* = 0.010 indicated as *). Data are mean and standard error.

Circulating concentrations of glucose, insulin, and triglycerides prior to and following food with placebo and CBD are presented in Figure 8, Figure 9 and Figure 10, respectively. CBD did not influence the glucose response to food (placebo vs. CBD × time interaction; *p* = 0.31) but did evoke lower insulin concentrations at minutes 10 and 20 (Placebo vs. CBD × time interaction; *p* = 0.013), and lower triglyceride concentrations at minute 30 (Placebo vs. CBD × time interaction; *p* = 0.010).

### 3.4. Influence of Food on the CBD Pharmacokinetics

**Figure 11:**
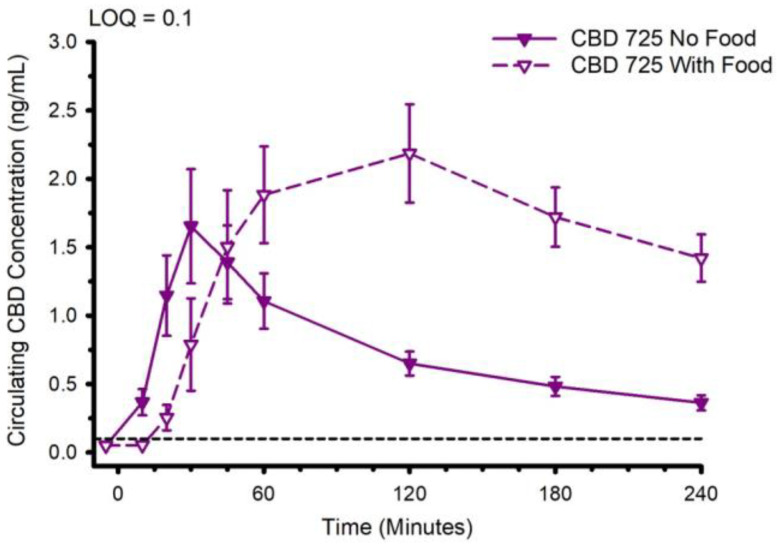
Circulating cannabidiol (CBD) concentration following ingestion of 30 mg of CBD with or without immediate prior ingestion of a mixed macronutrient meal. Abbreviation: LOQ Limit of Quantitation. Data are mean and standard error.

The CBD pharmacokinetics of formulation 725 (water-soluble, containing sorbitol) were compared when the CBD was ingested with and without prior consumption of food. The circulating CBD concentration is presented in Figure 11. Ingestion of CBD with prior consumption of food increased t_max_ from 38.2 ± 24.9 to 113.6 ± 70.5 min (*p* = 0.002), increased C_max_ from 1.8 ± 1.5 to 2.9 ± 1.3 ng/mL (*p* = 0.045), and increased AUC_0–4_ from 177 ± 104 to 397 ± 167 min × ng/mL (*p* < 0.001). With respect to the metabolites, ingestion of food typically slowed t_max_ and in some instances increased C_max_ and AUC_0–4_ (Supplementary Tables S2–S5).

### 3.5. Liver Function

Circulating markers of liver and kidney function are presented in Table 7. All baseline (pre-CBD) concentrations were considered normal. Compared with placebo, CBD had no appreciable effect on most of the markers. While several statistically significant effects were identified, the magnitudes of these changes were very modest and did not evoke concentrations outside of the thresholds for normal healthy recommended ranges. Changes in circulating concentrations of liver and kidney function markers were not related to circulating concentrations of CBD (all *p* > 0.05).

## 4. Discussion

The main findings of the current study were: (1) when delivered in an aqueous beverage, water-soluble CBD formulations typically evoked the fastest t_max_, and the greatest C_max_. (2) While CBD did not influence the thermic effect of food, it did lower circulating insulin and triglyceride concentrations during the first 30-min following food ingestion. (3) Consumption of food prior to CBD ingestion increased t_max_, C_max_ and AUC_0–4_. (4) Single 30 mg doses of CBD did not influence the majority of circulating markers of liver and kidney function. In the few markers that were increased, the magnitude of change was unlikely to be physiologically relevant.

A vast range of approaches have been explored for improving the systemic availability of CBD and augmenting CBD pharmacokinetics [5,6,7,11]. One approach that has received repeated attention pertains to the comparison of water- and lipid-soluble preparations [5,7,26]. Within the lipid-soluble preparations, various strategies have been further investigated that include the comparison of medium- and long-chain triglycerides, the rationale being that the latter will avoid first-pass hepatic metabolism and CBD will be absorbed via the intestinal lymphatic system [12,33]. Another complimentary approach has involved the use of self-emulsifying drug delivery systems (SEDDS) [37,38]. Both approaches have generated data that support the application of lipid-soluble preparations for use in ingestible, commercially available CBD products, such as gelatin capsules. An alternative and increasingly common method of commercial CBD consumption is via CBD-infused beverages and powders designed to be dissolved in water for co-consumption with teas, beers, and sodas [39,40]. Unless the beverages contain a relatively high fat content (e.g., whole milk) lipid-soluble CBD preparations are probably less appropriate for facilitating CBD delivery. In the current study, all the CBD preparations were delivered in 227 mL (8 oz) of water. Consistent with previous studies of CBD formulations prepared for use in beverages [5,26], the water-soluble preparations in the current study proved superior to the hydrophobic, lipid-soluble preparations in that they typically demonstrated faster t_max_, and greater C_max_ and AUC_0–4_. In particular, the formulation 126 (water-soluble, 10% CBD gum arabic mixture, tapioca maltodextrin base) returned a t_max_ of approximately 30 min; compared with previously published data [5,6], this would make it among the fastest of commercially available products. Maltodextrin is thought to help favorably modify osmotic pressure and promote transport from the intestine [35]. It has been used previously to improve the delivery of active ingredients in sports beverages [41,42]. It is plausible that inclusion of maltodextrin in the preparation of formulation 126 contributed to the fast CBD pharmacokinetics.

Other variables known to influence CBD pharmacokinetics include the co-administration of food. Several previous studies have described the ability of different types of food, particularly high-fat foods, to influence the CBD pharmacokinetics of a single dose of Epidiolex [8,9], a pharmaceutical grade CBD oral solution approved by the United States Food and Drug Administration for treatment of epilepsy. In two studies, co-consumption with food appreciably increased CBD C_max_ and AUC_0–inf_. Noteworthy, the doses of CBD in these studies were relatively high (750–1500 mg) and considerably greater than the CBD dose typically consumed from commercial products. The influence of food on CBD and CBD metabolite pharmacokinetics from lower dose commercial products is unknown. In our study, consistent with the Epidiolex trials, a mixed macronutrient meal increased t_max_, C_max_ and AUC_0–4_. Similarly, the ingestion of food typically slowed t_max_ and in some instances increased C_max_ and AUC_0–4_ for the CBD metabolites. Although we have no data to directly address potential mechanisms, we speculate that the meal-evoked secretion of digestive chemicals, such as bile, enhanced the absorption of CBD from the gut.

The thermic effect of food accounts for approximately 10% of total daily energy expenditure [43,44] and has been identified as a potential target for weight-loss intervention [22]. Several studies have reported on the lower thermic effect of food in adults with overweight and obesity [45], adults with a sedentary lifestyle [36,46], and in older compared with young adults [46,47]. Potential mechanisms previously purported to account for decreased thermogenic responsiveness and metabolic dysregulation in the described populations include, but are not limited to, chronic inflammation and oxidative stress [30,48,49,50]. In light of the anti-inflammatory and antioxidant properties of CBD [51], it appeared plausible that CBD might improve the thermic effect of feeding in adults with overweight and obesity. Our data, the first to address this question, do not support this line of thinking; CBD had negligible effects on energy expenditure and substrate utilization following food ingestion. However, CBD did evoke lower circulating concentrations of insulin and triglycerides during the first 30-min of the postprandial state. While a role of the endocannabinoid system in the regulation of glucose and blood lipids has been described [17], and CBD is thought to inhibit the actions of CBD receptors [20,21], data supporting a beneficial influence of CBD are mixed [16]. For example, in a variety of rat models, including high-fat diet and cerebral hypoperfusion, administration of CBD has been reported to both increase [52] and decrease [53] fasting insulin. In adult humans with type 2 diabetes, short-term (13-weeks) CBD administration (100 mg twice daily) had no effect on insulin [54]. Similarly, the influence of CBD on circulating triglycerides is also unclear, with studies of animals [52] and humans [54] reporting conflicting outcomes. Potential explanations for these inconsistent outcomes include differences in CBD dose, dosing duration and method of administration, differences in experimental/clinical disease models, and species differences. Pertinent to our study of adult humans with overweight and obesity, it may be important to note that the beneficial effects of CBD pertaining to insulin and triglycerides occurred early in the postprandial response when circulating concentrations of CBD and CBD metabolites were still relatively low (i.e., at a time well below t_max_). Thus, the CBD-mediated mechanism that evoked our observations is unclear. Nevertheless, the observations were consistent across most study participants and imply that CBD may favorably modify the early physiological responses to a mixed macronutrient meal.

The final aim of the current study pertained to the influence of CBD on markers of liver function. The endocannabinoid system is known to be involved with the development of multiple liver diseases, including cirrhosis, hepatitis and steatosis [55], and several studies have reported on adverse liver outcomes following CBD administration [25]. In addition, in clinical trials of Epidiolex, indicators of liver dysfunction/damage were noted in four reports [56,57,58,59]. Alternatively, in a recent cross-sectional study of 839 habitual CBD users, no association was identified between CBD and multiple markers of liver health and function [60]. Furthermore, based on a review of the potential contribution of the endocannabinoid system to liver disease, others have called for CBD to be included in comprehensive treatments for liver disorders [24]. In the current study, single 30 mg doses of CBD did not influence the majority of circulating markers of liver (and kidney) function. In the few markers that were increased, the magnitude of change was unappreciable, and the resultant concentrations were well within the limits of normalcy. Differences between our study and those reporting adverse liver outcomes include study sample size, dosing regimen (i.e., acute vs. short-term), and dosing size. With regard to the former, our study of 14 adults is considerably smaller than previous studies of hundreds [56,57,58,59], and thus our statistical outcomes should be interpreted with caution. With respect to dosing regimen and dosing size, these are potentially important differences, as the single 30 mg doses administered in the current study are appreciably lower than the short-term (weeks-to-months) daily doses of 20 mg per kg body mass administered in the Epidiolex clinical trials. It appears likely that non-clinical doses of commercially available CBD are unlikely to represent an appreciable risk to liver function. A similar sentiment was recently expressed in a large study of 839 healthy adults who regularly self-administered CBD (approximately 50 mg/day for 30 days); the authors speculated that the very few examples of unfavorable liver markers could be explained by co-existing medical conditions and/or the use of other supplements and medications [60].

### Limitations

Our original intention was to address our hypotheses in a population comprising adult males and females. However, somewhat unexpectedly, the protocol was only completed by males. While we have no reason to suspect the potential for sex differences, the extrapolation of our observations to females must be undertaken with a degree of caution. Furthermore, although our study incorporated a randomized, placebo-controlled, repeated-measures crossover design, the study population was relatively small (*n* = 14), making it plausible that the outcomes may have been different had the study been completed with a much larger sample size. In our thermic effect of food studies, in order to facilitate the standardization of the relative caloric load we utilized a liquid mixed macronutrient meal. Although commercially available meal replacements are becoming increasingly popular, they do not represent the type of meal most commonly ingested (i.e., solid foods). Indeed, differences in the thermic effect of unprocessed whole food-based meals and processed meal replacement shakes have previously been reported [61]. Future studies may incorporate more ecologically relevant food types; however, based on our data, we strongly suggest that CBD will not influence the thermic effect of ingesting whole/solid food meals. Finally, as alluded to in our discussion of data pertaining to liver function, we administered a single CBD dose that was appreciably smaller than that used in clinical trials of Epidiolex [56,57,58,59]. Indeed, the range in magnitude of the CBD dose that has been administered in human research is appreciable (e.g., 5–6000 mg) [5,6]. CBD appears to be safe and well-tolerated at low doses, but at higher doses commonly reported side effects include gastric distress, sleepiness, potentially inhibited immune function, and liver damage. There may also be potential for unfavorable interactions with prescribed medications. In a recent review of CBD safety [62], the authors reported on recommendations provided by the Committee on Toxicity of Chemicals in Food, Consumer Products and the Environment, a group of scientists who provide advice to branches of the UK government. The committee suggested that “a pragmatic upper level of intake above which there would be clear concerns about safety” was 1 mg of CBD per kg of body mass per day. We suggest that our studied dose of 30 mg is most relevant to the average 80 kg (176 lbs) consumer who self-administers over-the-counter CBD two-to-three times per day.

## 5. Conclusions

Consistent with previous studies, we have demonstrated considerable pharmacokinetic variability between different formulations of CBD standardized to 30 mg/dose. Furthermore, we have extended these data to demonstrate that this pharmacokinetic variability transfers to several of the CBD metabolites (6-OH-CBD, 7-OH-CBD, and CBD-COOH). In addition, we have shown for the first time that CBD does not influence the thermic effect of food in males with overweight and obesity, but it does favorably modify the initial insulin and triglyceride response. Food appears to appreciably alter the pharmacokinetics of CBD and CBD metabolites to increase systemic availability. Finally, we provide evidence to suggest that a single 30 mg dose of CBD does not appear to evoke physiologically relevant changes in markers of liver (and kidney) function.

## Figures and Tables

**Table 1:** Key features of each of the cannabidiol formulations.

Formulation	Description
725	Water soluble. Contains sorbitol.
088	Not water soluble. Contains medium chain triglyceride coconut oil.
126	Water soluble. Contains gum arabic and maltodextrin
213	Water soluble. Contains gum arabic and sorbitol
625	Not water soluble. Pure CBD as crystalline powder (>99% purity)

Each administered serving contained 30 mg of CBD. CBD, Cannabidiol.

**Table 2:** Selected physiological characteristics of study participants.

Variable	Mean ± SD	Range
Age (years)	26 ± 8	20–51
Height (cm)	176 ± 6	166–185
Body Mass (kg)	93.2 ± 12.8	72.8–120.9
Body Mass Index (kg/m^2^)	29.7 ± 2.8	25.8–35.2
Fat Mass (kg)	26.9 ± 65	15.4–37.4
% Body Fat	28.6 ± 4.7	20.7–38.0
Lean Mass (kg)	63.7 ± 8.3	52.2–80.4

Abbreviation: SD Standard deviation.

**Table 3:** Cannabidiol Pharmacokinetic Parameters.

Parameter		088	126	213	625	725
T_max_ (min)	Mean SD Median Range *n*	116.3 ^b,c,e^ 76.0 90.0 45–240 12	35.4 *^,b^ 13.5 30.0 20–60 14	51.8 ^d,e^ 24.2 52.5 20–120 14	129.5 *^,a,d^ 89.6 60 45–240 11	38.2 ^a,c^ 24.9 30.0 20–120 14
C_max_ (ng/mL)	Mean SD Median Range *n*	0.5 ^a,c^ 0.2 0.5 0.2–1.0 12	3.1 *^,a,e^ 2.1 2.8 0.8–7.1 14	2.2 ^b,c^ 2.0 1.5 0.5–7.8 14	0.4 *^,b,d^ 0.6 0.2 0.13–2.33 11	1.8 ^d,e^ 1.5 1.2 0.7–6.2 14
AUC_0–4_ (min × ng/mL)	Mean SD Median Range *n*	62.8 ^a,c,e^ 29.7 59.0 14.4–108.6 12	272.3 *^,a,f^ 176.0 238.1 82.5–602.5 14	208.6 ^b,c^ 151.1 153.0 67.6–582.9 14	46.0 *^,b,d^ 59.4 28.9 4.2–210.7 11	177.3 ^d,e,f^ 104.8 146.4 87.1–433.3 14
AUC_0–inf_ (min × ng/mL)	Mean SD Median Range *n*	- - - - -	385.2 226.6 319.6 93.8–676.0 6	367.6 217.1 285.6 163.7–704.1 5	- - - - -	301.6 221.9 301.6 144.7–458.5 2
t_½_ (min)	Mean SD Median Range *n*	280.7 141.5 245.7 151.5–523.4 5	171.0 129.2 113.2 55.4–464.9 14	140.5 96.6 106.4 80.0–415.9 11	442.7 451.0 200.7 164.2–963.1 3	133.1 26.7 128.7 94.6–181.8 13
k_e_ (1/h)	Mean SD Median *n*	0.003 0.001 0 5	0.006 0.003 0.010 14	0.006 0.002 0.01 11	0.003 0.002 0.000 3	0.005 0.001 0.010 13
V_d_ (mL)	Mean SD Median *n*	- - - -	11836405 7642484 11901025 6	13130100 6339554 14091985 5	- - - -	22687960 16435999 22687960 2

SD: Standard Deviation. Limit of quantitation: 0.1 ng/mL. Values below limit of quantitation were classed as “missing”. *n*: number of observations used to calculate parameter. T_max_: the time to maximum concentration. C_max_: the maximum concentration. AUC_0–4_: the area under the curve representing total cannabidiol exposure between 0 and 4 h. AUC_0–inf_: an estimate of the total exposure to cannabidiol over time. t_½_: the amount of time it takes to decrease the circulating concentration to half of its initial value. k_e_: the rate at which the cannabidiol is removed from the body. V_d_: the volume of distribution, an estimate of the degree to which cannabidiol is distributed in the body tissue vs. the plasma. Values sharing the same superscript letter or symbol are different (*p* < 0.05).

**Table 4:** 6-OH-Cannabidiol Pharmacokinetic Parameters.

Parameter		088	126	213	625	725
T_max_ (min)	Mean SD Median Range *n*	120.0 ^c,d,e^ 69.3 120.0 60–240 7	45.0 *^,c^ 13.2 45.0 30–60 14	66.4 ^b,e^ 30.4 60.0 30–120 14	144.0 *^,a,b^ 91.0 120.0 60–240 5	50.4 ^a,d^ 30.9 45.0 30–120 14
C_max_ (ng/mL)	Mean SD Median Range *n*	0.2 *^,c,d^ 0.1 0.2 0.1–0.3 7	0.6 *^,a,b^ 0.3 0.5 0.3–1.5 14	0.5 ^c^ 0.4 0.3 0.2–1.8 14	0.2 ^a,e^ 0.1 0.2 0.1–0.4 5	0.4 ^b,d,e^ 0.2 0.4 0.1–0.9 14
AUC_0–4_ (min × ng/mL)	Mean SD Median Range *n*	18.0 *^,c,e^ 9.4 16.6 3.4–31.3 7	65.1 *^,a,b^ 49.0 52.1 9.3–209.7 14	51.3 ^c,d^ 40.6 40.3 6.7–157.1 14	18.4 ^a,d,f^ 11.9 16.0 6.2–37.7 5	45.3 ^b,e,f^ 27.0 44.2 5.7–90.3 14
AUC_0–inf_ (min × ng/mL)	Mean SD Median Range *n*	- - - - -	240.3 - 240.3 240.3 1	126.6 68.7 126.6 78.0–175.2 2	- - - - -	- - - - -
t_½_ (min)	Mean SD Median Range *n*	- - - - -	175.1 141.4 118.7 68.8–543.3 11	195.0 130.7 143.2 62.6–380.22 7	- - - - -	186.7 88.3 161.4 81.0–390.0 10
K_e_ (1/h)	Mean SD Median *n*	- - - -	0.006 0.003 0.010 11	0.005 0.004 0.000 7	- - - -	0.004 0.002 0.000 10
V_d_ (mL)	Mean SD Median *n*	- - - -	12396095 - 12396095 1	31161801 22199818 31161801 2	- - - -	- - - -

See Table 3 and manuscript text for abbreviation definitions. Values sharing the same superscript letter or symbol are different (*p* < 0.05).

**Table 5:** 7-OH-Cannabidiol Pharmacokinetic Parameters.

Parameter		088	126	213	625	725
T_max_ (min)	Mean SD Median Range *n*	110.4 ^c,d,e,f^ 58.1 120 45–240 14	60.0 ^b,f^ 42.0 52.5 30–180 14	58.9 ^a,e^ 19.9 60 30–120 14	156.9 *^,a,b,c^ 67.3 120.0 60–240 13	55.7 *^,d^ 20.7 52.5 30–120 14
C_max_ (ng/mL)	Mean SD Median Range *n*	0.62 ^a,e,g^ 0.38 0.54 0.2–1.3 14	4.89 *^,a,b,c^ 3.78 3.91 1.8–16.0 14	3.14 ^c,d,e^ 1.82 2.48 0.8–6.4 14	0.71 *^,d,f^ 1.45 0.26 0.1–5.5 13	2.73 ^b,f,g^ 1.64 2.27 1.0–6.3 14
AUC_0–4_ (min × ng/mL)	Mean SD Median Range *n*	88.8 ^a,e,g^ 53.1 84.4 22.0–172.2 14	523.6 *^,a,b,c^ 350.1 392.6 184.3–1389.7 14	380.1 ^c,d,e^ 208.7 295.7 100.9–782.7 14	81.0 *^,d,f^ 132.5 43.0 8.0–506.3 13	343.9 ^b,f,g^ 174.3 292.0 139.2–706.0 14
AUC_0–inf_ (min × ng/mL)	Mean SD Median Range *n*	- - - -	598.2 391.7 487.8 216.9–1244.1 5	584.1 208.7 586.4 314.3–893.0 5	- - - -	531.1 207.1 455.1 372.8–765.5 3
t_½_ (min)	Mean SD Median Range *n*	368.9 407.9 200.4 98.9–975.9 4	108.1 38.6 94.8 62.5–177.6 12	132.4 75.9 117.7 70.1–360.3 13	163.6 57.6 163.6 122.8–204.3 2	109.6 25.0 104.4 78.2–166.8 13
K_e_ (1/h)	Mean SD Median *n*	0.004 0.003 0.000 4	0.007 0.002 0.010 12	0.006 0.002 0.010 13	0.005 0.002 0.000 2	0.007 0.001 0.010 13
V_d_ (mL)	Mean SD Median *n*	- - - -	7361772 4728622 6678818 5	6506682 2353415 6593583 5	- - - -	8096154 3862326 7746967 3

See Table 3 and manuscript text for abbreviation definitions. Values sharing the same superscript letter or symbol are different (*p* < 0.05).

**Table 6:** COOH-Cannabidiol Pharmacokinetic Parameters.

Parameter		088	126	213	625	725
T_max_ (min)	Mean SD Median Range n	175.7 ^c,d,e,f^ 49.7 180.0 120–240 14	93.2 *^,d^ 40.0 90.0 45–180 14	111.4 ^b,f^ 32.1 120.0 60–180 14	210.0 *^,a,b,c^ 45.6 240.0 120–240 14	111.4 ^a,e^ 32.1 120.0 60–180 14
C_max_ (ng/mL)	Mean SD Median Range n	15.6 ^a,f^ 9.1 14.4 3.6–33.4 14	86.7 *^,a,b,c^ 58.2 72.3 21.1–249.7 14	59.3 ^c,d^ 29.8 51.4 11.7–118.7 14	12.2 *^,d,e^ 11.8 7.4 1.2–42.9 14	53.8 ^b,e,f^ 22.9 51.6 13.4–108.4 14
AUC_0–4_ (min × ng/mL)	Mean SD Median Range n	2284.9 ^a,e,g^ 1361.2 2276.1 547–5275 14	14907.4 *^,a,b,c^ 10354.6 12327.3 3778–43275 14	10233.4 ^c,d,e^ 5421.8 8900.2 2127–23257 14	1743.7 *^,d,f^ 1907.4 974.0 183–7126 14	9457.3 ^b,f,g^ 4263.4 7923.3 2432–20208 14
AUC_0–inf_ (min × ng/mL)	Mean SD Median Range n	- - - - -	- - - - -	- - - - -	- - - - -	- - - - -
t_½_ (min)	Mean SD Median Range n	- - - - -	408.6 292.0 296.4 154.9–981.1 7	385.8 150.1 431.4 218.2–507.9 3	- - - - -	282.5 26.6 267.8 266.4–313.2 3
K_e_ (1/h)	Mean SD Median n	- - - -	0.002 0.001 0.000 7	0.002 0.001 0.000 3	- - - -	0.002 0.000 0.000 3
V_d_ (mL)	Mean SD Median n	- - - -	- - - -	- - - -	- - - -	- - - -

See Table 3 and manuscript text for abbreviation definitions. Values sharing the same superscript letter or symbol are different (*p* < 0.05).

**Table 7:** Circulating markers of liver and kidney function prior to and following the ingestion of CBD.

Formula	Time min	Ca mg/dL	BUN mg/dL	CRE mg/dL	ALP U/L	ALT U/L	AST U/L	TBIL mg/dL	ALB g/dL	TP g/dL
Placebo	0	9.4	15.1	1.1	62.1	26.1	28.0	0.8	3.6	6.5
		±0.6	±2.7	±0.2	±8.6	±10.1	±12.3	±0.3	±0.3	±0.5
	60	9.4	14.6	1.1	62.9	26.9	30.7	0.8	3.6	6.6
		±0.5	±2.9	±0.2	±9.8	±10.8	±10.4	±0.3	±0.2	±0.5
	240	9.3	13.9	1.1	58.8	26.0	29.4	0.8	3.4	6.3
		±0.5	±2.5	±0.3	±8.5	±11.2	±10.1	±0.3	±0.3	±0.4
625	0	9.3	15.0	1.1	61.9	30.5	30.6	0.9	3.8	6.7
		±0.5	±3.6	±0.2	±9.4	±11.2	±7.3	±0.2	±0.3	±0.6
	60	9.5	14.8	1.1	61.6	30.4	31.4	0.9	3.8	6.8
		±0.4	±3.9	±0.3	±9.3	±11.0	±7.4	±0.2	±0.2	±0.4
	240	9.1	13.8	1.1	62.3	30.7	31.9	1.0	4.0	7.0
		±2.2	±3.7	±0.2	±9.2	±11.5	±7.5	±0.3	±0.2	±0.7
088	0	8.9	14.6	1.1	62.5	28.0	30.5	1.0	3.9	6.8
		±2.2	±2.8	±0.2	±7.9	±10.9	±4.1	±0.3	±0.3	±0.3
	60	9.6	14.4	1.2	60.5	27.9	30.6	0.9	3.8	6.7
		±0.4	±2.8	±0.2	±7.2	±10.0	±4.6	±0.2	±0.3	±0.4
	240	9.7	13.4	1.2	60.2	28.6	31.1	1.0	3.9	6.9
		±0.5	±2.5	±0.2	±8.0	±10.6	±6.8	±0.2	±0.3	±0.6
213	0	9.3	15.4	1.2	60.3	29.0	33.5	1.0	3.8	6.7
		±0.5	±3.9	±0.2	±6.8	±10.6	±7.0	±0.4	±0.3	±0.5
	60	9.4	15.5	1.1	61.0	29.1	30.9	1.0	3.8	6.7
		±0.4	±4.1	±0.4	±8.7	±9.8	±5.1	±0.4	±0.2	±0.3
	240	9.5	14.3	1.1	62.1	28.7	30.4	1.1	3.9	6.8
		±0.3	±3.5	±0.3	±9.5	±10.5	±5.9	±0.4	±0.2	±0.3
126	0	9.4	15.4	1.1	63.6	27.5	30.2	1.0	3.9	6.8
		±0.3	±4.5	±0.2	±10.8	±8.4	±3.4	±0.6	±0.2	±0.2
	60	9.5	15.1	1.2	61.9	29.3	29.4	1.0	3.8	6.7
		±0.5	±4.6	±0.3	±11.0	±8.1	±8.7	±0.6	±0.3	±0.4
	240	9.5	14.0	1.1	61.4	28.2	30.3	1.1	3.9	6.8
		±0.4	±4.2	±0.3	±9.6	±7.9	±4.1	±0.6	±0.3	±0.3
725	0	9.4	15.9	1.1	60.4	27.7	29.2	0.9	3.8	6.8
		±0.4	±3.3	±0.3	±12.0	±9.1	±5.0	±0.4	±0.3	±0.4
	60	9.6	15.3	1.1	60.9	27.3	28.9	0.9	3.8	6.7
		±0.2	±3.3	±0.1	±11.4	±8.7	±4.3	±0.4	±0.2	±0.3
	240	8.9	14.1	1.0	61.0	27.6	29.6	1.0	3.9	6.9
		±2.3	±3.1	±0.2	±12.6	±9.8	±4.4	±0.4	±0.2	±0.4
*p*-Value	CBD	0.964	0.765	0.836	0.863	0.462	0.810	0.022	<0.001	0.010
	Time	0.370	<0.001	0.483	0.480	0.425	0.979	<0.001	0.052	0.291
	Inter.	0.417	0.368	0.163	0.358	0.614	0.230	<0.001	0.002	0.058

Data are mean ± standard deviation. Each cannabidiol serving contained 30 mg of cannabidiol. *p*-Values are derived from two-way analysis of variance (formula × time) with repeated measures (time). All comparisons: *n* = 14. Ca: Calcium. BUN: Blood urea nitrogen. CRE: Creatinine. ALP: Alkaline phosphatase. ALT: Alanine aminotransferase. AST: Aspartate aminotransferase. TBIL: Total Bilirubin. ALB: Albumin. TP: Total protein. Inter: Statistical interaction between CBD formulation and time. CBD: cannabidiol.

## Data Availability

The data presented in this study are available on request from the corresponding author.

## Supplementary Materials

The following supporting information can be downloaded at: https://www.mdpi.com/article/10.3390/nu14102152/s1, Figure S1: Heart rate prior to and following ingestion of food with/without cannabidiol (CBD). Figure S2. Blood pressure prior to and following ingestion of food with/without cannabidiol (CBD). Table S1: LC-MS/MS ion transitions monitored for dansyl-derivatives of CBD and metabolites in human plasma. Table S2: Influence of Eating On Cannabidiol Pharmacokinetic Parameters. Table S3. Influence of Eating On 6-OH-Cannabidiol Pharmacokinetic Parameters. Table S4. Influence of Eating On 7-OH-Cannabidiol Pharmacokinetic Parameters. Table S5. Influence of Eating On COOH-Cannabidiol Pharmacokinetic Parameters
