# Supplementary material for: Cannabidiol and Cannabidiol Metabolites: Pharmacokinetics, Interaction with Food, and Influence on Liver Function
Source: Nutrients. 2022 May 21;14(10):2152. doi: 10.3390/nu14102152 (PMC9144241; doi:10.3390/nu14102152)
Supplement: Supplementary file 1 [file nutrients-14-02152-s001.zip › Supplementary Table S1.pdf]

Table S1. LC-MS/MS ion transitions monitored for dansyl-derivatives of CBD and metabolites in human plasma.

Analyte Name	Precursor Ion	Product Ion	Frag (V)	CE (V)	Cell Acc (V)	Polarity
Dansyl-7-carboxy-CBD	811.3	796.2	130	24	2	Positive
Dansyl-7-carboxy-CBD	811.3	170.1	130	45	2	Positive
Dansyl-7-carboxy-CBD-D3	814.3	793.2	130	24	2	Positive
Dansyl-7-carboxy-CBD-D3	814.3	170.1	130	45	2	Positive
Dansyl-7-hydroxy-CBD	797.3	779.3	80	8	2	Positive
Dansyl-7-hydroxy-CBD	797.3	17.01	80	45	2	Positive
Dansyl-7-hydroxy-CBD-D3	800.4	782.3	80	8	2	Positive
Dansyl-7-hydroxy-CBD-D3	800.4	170.1	80	45	2	Positive
Dansyl-6-hydroxy-CBD	779.3	545.2	165	24	2	Positive
Dansyl-6-hydroxy-CBD	779.3	170.1	165	45	2	Positive
Dansyl-CBD	781.3	156	205	28	2	Positive
Dansyl-CBD	781.3	170.1	205	45	2	Positive
Dansyl-CBD-D3	784.3	535.2	205	28	2	Positive
Dansyl-CBD-D3	784.3	170.1	205	45	2	Positive
