# Supplementary material for: Cannabidiol and Cannabidiol Metabolites: Pharmacokinetics, Interaction with Food, and Influence on Liver Function
Source: Nutrients. 2022 May 21;14(10):2152. doi: 10.3390/nu14102152 (PMC9144241; doi:10.3390/nu14102152)
Supplement: Supplementary file 1 [file nutrients-14-02152-s001.zip › Supplementary Table S2.pdf]

Table S2. Influence of Eating On Cannabidiol Pharmacokinetic Parameters

Parameter		725 No Food	725 + Food
T_{max} * (min)	Mean	38.2	113.6
	SD	24.9	70.5
	Median	30.0	90.0
	Range	20 – 120	45 – 240
	n	14	14
C_{max} * (ng/mL)	Mean	1.8	2.9
	SD	1.5	1.3
	Median	1.2	2.9
	n	14	14
AUC_{0-4} * (min x ng/mL)	Mean	177.3	397.1
	SD	104.8	167.2
	Median	146.4	412.5
	n	14	14
AUC_{0-inf} (min x ng/mL)	Mean	301.6	-
	SD	221.9	-
	Median	301.6	-
	n	2	-
$t_{½}$ (min)	Mean	133.1	248.6
	SD	26.7	304.6
	Median	128.7	126.6
	n	13	7
K_e (1/hr)	Mean	0.005	0.005
	SD	0.001	0.002
	Median	0.010	0.010
	n	13	7
V_d (mL)	Mean	22687960	-
	SD	16435999	-
	Median	22687960	-
	n	2	-

SD: Standard Deviation. Limit of quantitation: 0.1 ng/mL. Food was a commercially available mixed macronutrient liquid meal (22% fat, 62% carbohydrate, 16% protein); caloric equivalent to 40% of resting metabolic rate. Values below limit of quantitation were classed as “missing”. *n*: number of observations used to calculate parameter. T_{max}: the time to maximum concentration. C_{max}: the maximum concentration. AUC_{0-4}: the area under the curve representing total cannabidiol exposure between 0 and 4 hours. AUC_{0-inf}: an estimate of the total exposure to cannabidiol over time. $t_{½}$: the amount of time it takes to decrease the circulating concentration to half of its initial value. K_e: the rate at which the cannabidiol is removed from the body. V_d: the

volume of distribution, an estimate of the degree to which cannabidiol is distributed in the body tissue *vs*. the plasma. Parameters marked with * are different ($P < 0.05$).
