# Supplementary material for: Cannabidiol and Cannabidiol Metabolites: Pharmacokinetics, Interaction with Food, and Influence on Liver Function
Source: Nutrients. 2022 May 21;14(10):2152. doi: 10.3390/nu14102152 (PMC9144241; doi:10.3390/nu14102152)
Supplement: Supplementary file 1 [file nutrients-14-02152-s001.zip › Supplementary Table S3.pdf]

Table S3. Influence of Eating On 6-OH-Cannabidiol Pharmacokinetic Parameters

Parameter		725 No Food	725 + Food
T_{max} * (min)	Mean	50.4	147.7
	SD	30.9	67.6
	Median	45.0	120
	Range	30 – 120	60 – 240
	n	14	13
C_{max} * (ng/mL)	Mean	0.4	0.3
	SD	0.2	0.2
	Median	0.4	0.2
	n	14	13
AUC_{0-4} (min x ng/mL)	Mean	45.3	39.8
	SD	27.0	26.4
	Median	44.2	32.7
	n	14	13
AUC_{0-inf} (min x ng/mL)	Mean	-	-
	SD	-	-
	Median	-	-
	n	-	-
$t_{½}$ (min)	Mean	186.7	221.1
	SD	88.3	86.3
	Median	161.4	221.1
	n	10	2
K_e (1/hr)	Mean	0.004	0.003
	SD	0.002	0.001
	Median	0.000	0.000
	n	10	2
V_d (mL)	Mean	-	-
	SD	-	-
	Median	-	-
	n	-	-

SD: Standard Deviation. Limit of quantitation: 0.1 ng/mL. Food was a commercially available mixed macronutrient liquid meal (22% fat, 62% carbohydrate, 16% protein); caloric equivalent to 40% of resting metabolic rate. Values below limit of quantitation were classed as "missing". *n*: number of observations used to calculate parameter. T_{max}: the time to maximum concentration. C_{max}: the maximum concentration. AUC_{0-4}: the area under the curve representing total 6-OH-cannabidiol exposure between 0 and 4 hours. AUC_{0-inf}: an estimate of the total exposure to 6-OH-cannabidiol over time. $t_{½}$: the amount of time it takes to decrease the circulating concentration to half of its initial value. K_e: the rate at which the 6-OH-cannabidiol is removed from the body. V_d: the volume of distribution, an estimate of the degree to which 6-OH-

cannabidiol is distributed in the body tissue *vs*. the plasma. Parameters marked with * are different ($P < 0.05$).
