# Supplementary material for: Cannabidiol and Cannabidiol Metabolites: Pharmacokinetics, Interaction with Food, and Influence on Liver Function
Source: Nutrients. 2022 May 21;14(10):2152. doi: 10.3390/nu14102152 (PMC9144241; doi:10.3390/nu14102152)
Supplement: Supplementary file 1 [file nutrients-14-02152-s001.zip › Supplementary Table S4.pdf]

Table S4. Influence of Eating On 7-OH-Cannabidiol Pharmacokinetic Parameters

Parameter		725 No Food	725 + Food
T_{max} * (min)	Mean	55.7	162.9
	SD	20.7	64.1
	Median	52.5	180
	Range	30 – 120	60 – 240
	n	14	14
C_{max} (ng/mL)	Mean	2.73	1.93
	SD	1.64	0.83
	Median	2.27	1.8
	n	14	14
AUC_{0-4} (min x ng/mL)	Mean	343.9	313.3
	SD	174.3	147.9
	Median	292.0	296.9
	n	14	14
AUC_{0-inf} (min x ng/mL)	Mean	531.1	-
	SD	207.1	-
	Median	455.1	-
	n	3	-
$t_{½}$ (min)	Mean	109.6	438.3
	SD	25.0	284.9
	Median	104.4	438.3
	n	13	2
K_e (1/hr)	Mean	0.007	0.002
	SD	0.001	0.001
	Median	0.010	0.000
	n	13	2
V_d (mL)	Mean	8096154	-
	SD	3862326	-
	Median	7746967	-
	n	3	-

SD: Standard Deviation. Limit of quantitation: 0.1 ng/mL. Food was a commercially available mixed macronutrient liquid meal (22% fat, 62% carbohydrate, 16% protein); caloric equivalent to 40% of resting metabolic rate. Values below limit of quantitation were classed as "missing". *n*: number of observations used to calculate parameter. T_{max}: the time to maximum concentration. C_{max}: the maximum concentration. AUC_{0-4}: the area under the curve representing total 7-OH-cannabidiol exposure between 0 and 4 hours. AUC_{0-inf}: an estimate of the total exposure to 7-OH-cannabidiol over time. $t_{½}$: the amount of time it takes to decrease the circulating concentration to half of its initial value. K_e: the rate at which the 7-OH-cannabidiol is removed from the body. V_d: the volume of distribution, an estimate of the degree to which 7-OH-

cannabidiol is distributed in the body tissue *vs*. the plasma. Parameters marked with * are different ($P < 0.05$).
