# Supplementary material for: Cannabidiol and Cannabidiol Metabolites: Pharmacokinetics, Interaction with Food, and Influence on Liver Function
Source: Nutrients. 2022 May 21;14(10):2152. doi: 10.3390/nu14102152 (PMC9144241; doi:10.3390/nu14102152)
Supplement: Supplementary file 1 [file nutrients-14-02152-s001.zip › Supplementary Table S5.pdf]

Table S5. Influence of Eating On COOH-Cannabidiol Pharmacokinetic Parameters

Parameter		725 No Food	725 + Food
T_{max} * (min)	Mean	111.4	201.4
	SD	32.1	55.7
	Median	120.0	240.0
	Range	60 – 180	60 – 240
	n	14	14
C_{max} * (ng/mL)	Mean	53.8	35.0
	SD	22.9	19.4
	Median	51.6	32.4
	n	14	14
AUC_{0-4} * (min x ng/mL)	Mean	9457.3	5116.7
	SD	4263.4	2429.0
	Median	7923.3	5641.1
	n	14	14
AUC_{0-inf} (min x ng/mL)	Mean	-	-
	SD	-	-
	Median	-	-
	n	-	-
$t_{½}$ (min)	Mean	282.5	830.2
	SD	26.6	-
	Median	267.8	830.2
	n	3	1
K_e (1/hr)	Mean	0.002	0.001
	SD	0.000	-
	Median	0.000	0.000
	n	3	1
V_d (mL)	Mean	-	-
	SD	-	-
	Median	-	-
	n	-	-

SD: Standard Deviation. Limit of quantitation: 0.1 ng/mL. Food was a commercially available mixed macronutrient liquid meal (22% fat, 62% carbohydrate, 16% protein); caloric equivalent to 40% of resting metabolic rate. Values below limit of quantitation were classed as “missing”. *n*: number of observations used to calculate parameter. T_{max}: the time to maximum concentration. C_{max}: the maximum concentration. AUC_{0-4}: the area under the curve representing total COOH-cannabidiol exposure between 0 and 4 hours. AUC_{0-inf}: an estimate of the total exposure to COOH-cannabidiol over time. $t_{½}$: the amount of time it takes to decrease the circulating COOH-concentration to half of its initial value. K_e: the rate at which the COOH-cannabidiol is removed from the body. V_d: the volume of distribution, an estimate of the degree

to which COOH-cannabidiol is distributed in the body tissue *vs*. the plasma. Parameters marked with * are different ($P < 0.05$).
