# Supplementary figures and images for: Cannabidiol and Cannabidiol Metabolites: Pharmacokinetics, Interaction with Food, and Influence on Liver Function
Source: Nutrients. 2022 May 21;14(10):2152. doi: 10.3390/nu14102152 (PMC9144241; doi:10.3390/nu14102152)

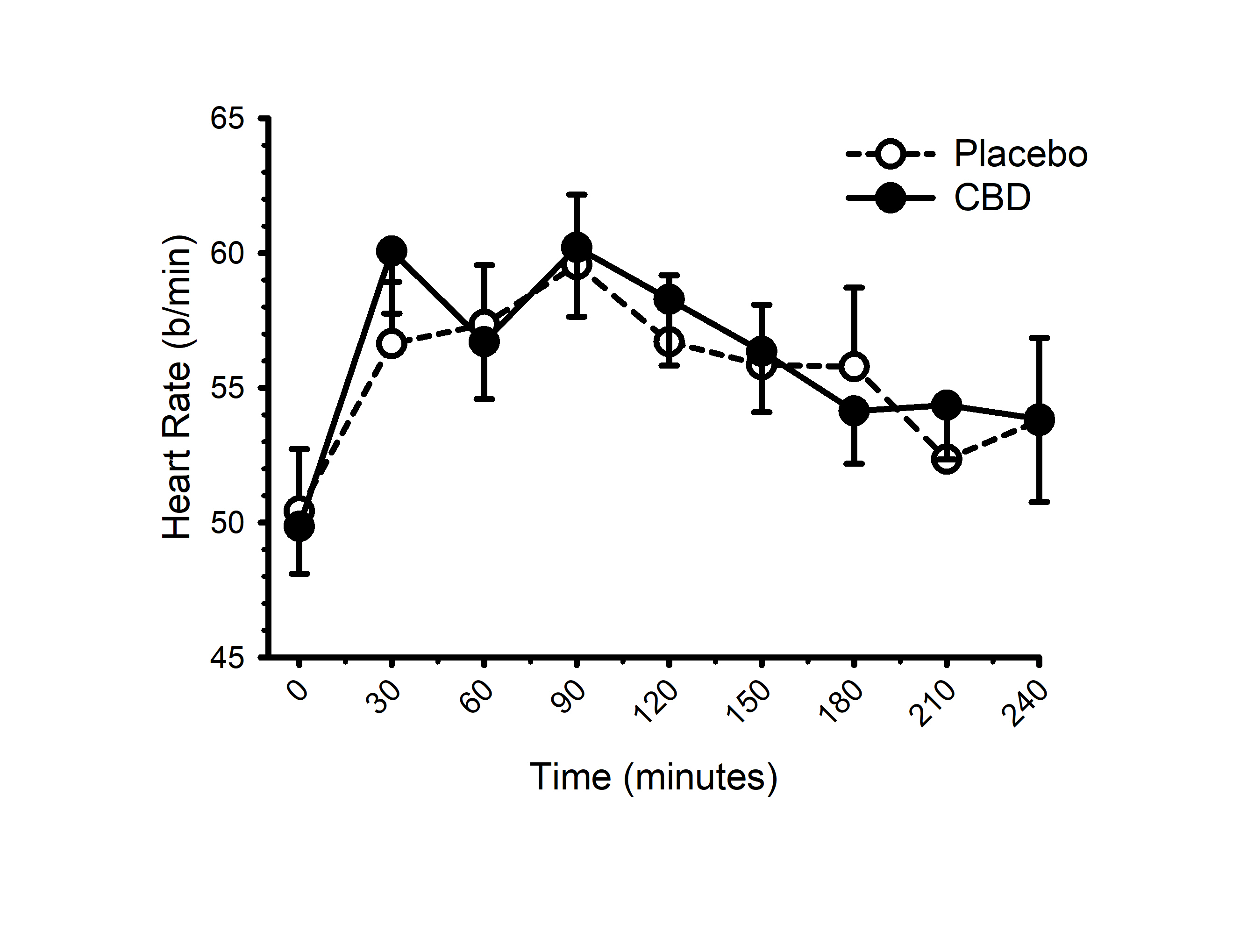

Supplement: Supplementary file 1 [file nutrients-14-02152-s001.zip › Supplementary Figure 1.JPG]

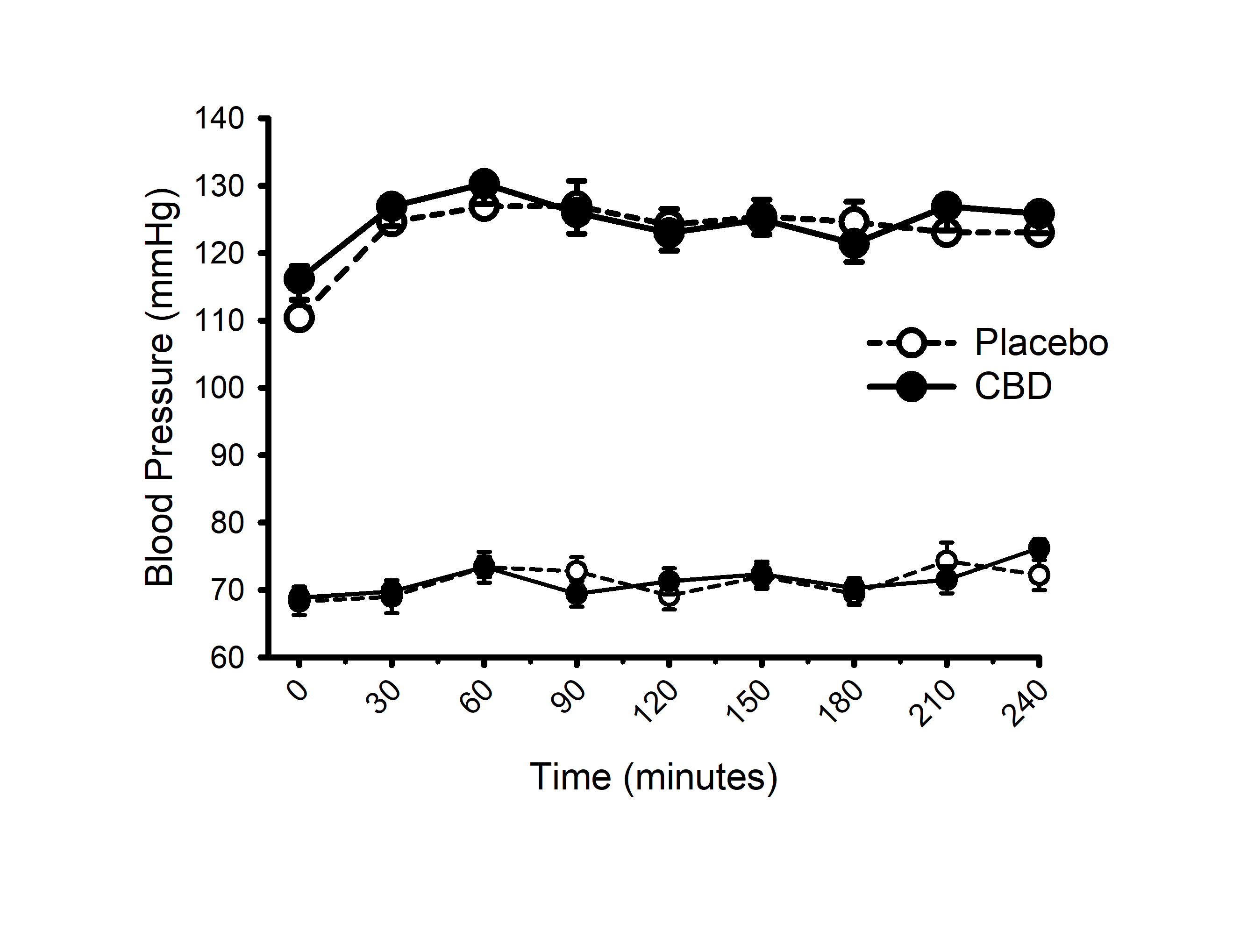

Supplement: Supplementary file 1 [file nutrients-14-02152-s001.zip › Supplementary Figure 2.JPG]
